# Unique features of non-compaction of the ventricular myocardium in Arab and African patients

**Published:** 2008-10

**Authors:** Sulafa KM Ali

**Affiliations:** Department of Pediatric Cardiology, Sudan Heart Centre, Khartoum, Sudan

## Abstract

**Summary:**

Non-compaction of the ventricular myocardium (NCVM) is an under-diagnosed cardiomyopathy. Patients diagnosed with NCVM at the King Abdulaziz Cardiac Centre, Riyadh, KSA from January 2000 to July 2004 and at the Sudan Heart Centre from August 2004 to July 2007 were included. Fifty-two patients with NCVM were identified (22 per 10 000 echocardiograms). Patients were divided into three groups, namely, group 1: isolated NCVM (21 patients), group 2: NCVM associated with congenital heart disease (CHD) (26 patients), and group 3: NCVM associated with mitral regurgitation (MR) (seven patients).

Group 1 included 14 females and four males. Five patients (27%) had a positive family history with a lethal outcome in five other siblings; 14 patients (76%) presented with myocardial dysfunction and two had left ventricle thrombus. Group 2 included CHD; the most common pathologies were ventricular septal defects (VSD), pulmonary and tricuspid atresia and hypoplastic left heart syndrome. Sixteen patients (61%) had myocardial dysfunction, seven had surgical repair/palliation, and four (80%) developed serious post-operative complications. Group 3 included seven patients with MR associated with deformity of the anterior mitral leaflet and malcoaptation. Myocardial function was preserved in all patients with this pathology. In four patients of the whole cohort there was clinical as well as echocardiographic improvement. In two patients, left ventricular hypertrophy was noted. There were significantly more females in the group with isolated NCVM than in the group with associated CHD (*p* = 0.03, odds ratio = 4.2, 95% CI = 0.529−16.1).

We presented the largest series of NCVM in our area and found it to be not as rare as was thought, with females being more affected. Spontaneous improvement and left ventricular hypertrophy were unique features, and mitral valve deformity leading to MR was an established association.

## Summary

Non-compaction of the ventricular myocardium is a cardiomyopathy characterised by excessive myocardial trabeculations and deep inter-trabecular recesses. It can be either isolated or associated with a range of heart diseases including simple and complex congenital heart disease (CHD). We also observed a unique association between NCVM and mitral valve abnormalities leading to significant mitral regurgitation (MR).^1^ This disease is thought to be under-diagnosed, as has been observed by us and others.^2^,^3^ We are reporting on our experience with non-compaction in two large centres from Saudi Arabia and Sudan where we observed a relatively high frequency and some unique features.

## Patients and methods

All patients diagnosed with non-compaction of the ventricular myocardium at King Abdulaziz Cardiac Centre, Riyadh, Kingdom of Saudi Arabia (KSA) from January 2000 to July 2004 and at the Sudan Heart Centre (SHC) from August 2004 to July 2007 were prospectively followed up. Patients were evaluated clinically and by complete two-dimensional echocardiography using HP 5500 (KSA) and MEGAS, Esotate (Sudan) echocardiography machines. We followed up on patients two to six monthly by clinical and echocardiography examination, according to their diagnosis.

For a diagnosis of non-compaction, using strict echocardiographic criteria in all patients, the following were adhered to:^4^,^5^

● measurement of the non-compacted-to-compacted layer thickness ratio of more than 2:1 from the parasternal short-axis view distal to the papillary muscles at the end of systole
● demonstration of the inter-trabecular recesses using lowscale colour-flow mapping
● identification of a distinct two-layer appearance.

We also examined the patients carefully for associated congenital or acquired heart disease, particularly mitral valve disease. The ejection fraction was measured in each study.

The data were analysed using the statistical package for social sciences (SPSS). Ethical approval was obtained from the ethics committees of the centres involved.

## Results

During the study period, 52 patients were diagnosed with non-compaction, 30 patients from KSA and 22 from SHC. All patients seen in KSA were Arabs whereas two of the Sudanese patients were Arabs and 17 were Arab/African. In this period, 13 600 echocardiograms were performed at the King Absulaziz Centre and 4 500 at the SHC, so the frequency of non-compaction was 22 per 10 000 echocardiograms in both centres. The patients were divided into the following three groups.

## Group 1: isolated non-compaction

**Fig. 1.**
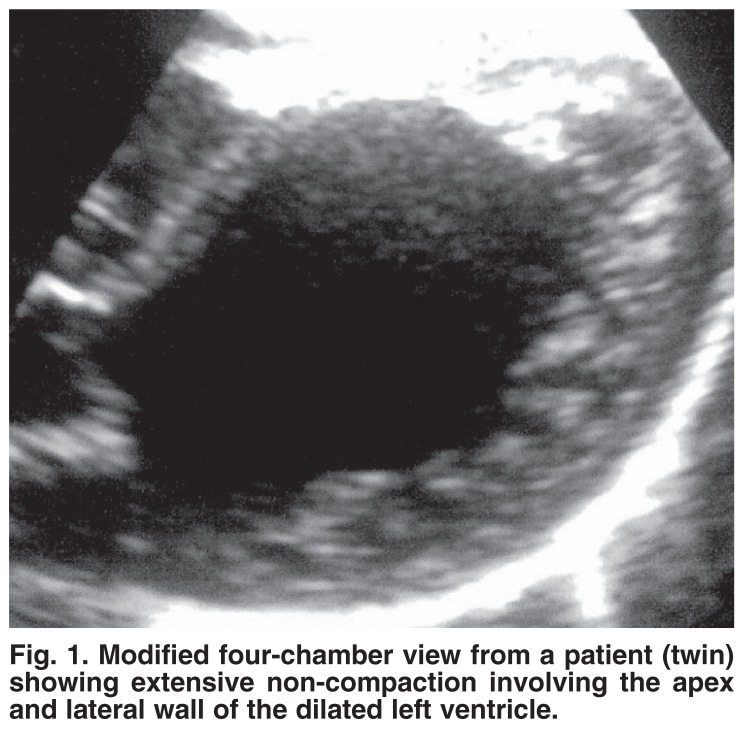
Modified four-chamber view from a patient (twin) showing extensive non-compaction involving the apex and lateral wall of the dilated left ventricle.

Twenty-one patients, 16 females and four males, had isolated NCVM (Table 1). Sixteen patients (76%) presented with heart failure due to depressed myocardial function. One was screened because of multiple congenital anomalies, two parents were screened because of an affected daughter, one twin sister was screened because of her affected twin (Fig. 1) and one presented with supraventricular tachycardia. Five patients (23%) had a positive family history, including two pairs of female twins. Six other children (not included in the study), five of whom were females, were affected.

**Table 1:** Clinical Features Of Patients With Isolated Non-Compaction (*N* = 21)

Age in months range (mean)	0−540 (73)
Male/female ratio	1:4
Asymptomatic	4 (19)
Heart failure with myocardial dysfunction (%)	16 (76)
Arrhythmia at presentation (%)	3 (14)
Improvement of myocardial dysfunction (%)	2 (9)
Death (%)	2 (9)
Family history (%)	5 (23)
Thrombo-embolism (%)	2 (9)
Associated extracardiac abnormalities (%)	2 (9)

**Fig. 2.**
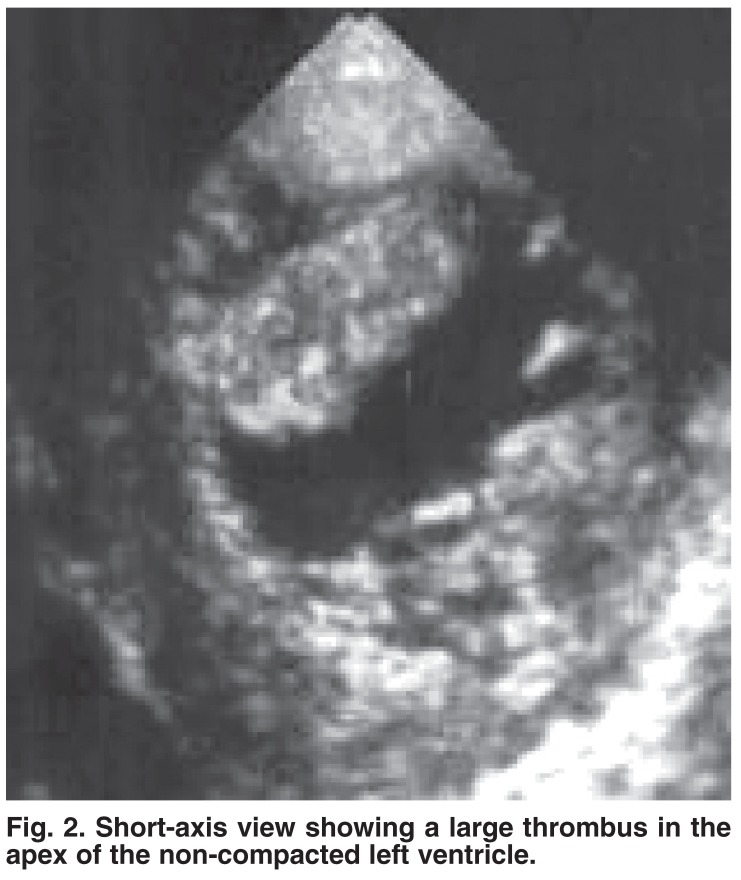
Short-axis view showing a large thrombus in the apex of the non-compacted left ventricle.

Associated extra-cardiac anomalies included severe hypotonia in one family and hydrocephalus with Dandy-Walker syndrome in one patient. Two patients developed left ventricle thrombus (Fig. 2), one of whom had right-sided hemiparesis that improved over three days. Two patients (9%) died; the causes of death were ventricular arrhythmia (one patient) and severe heart failure associated with hypotonia (one patient). Most other patients continued to have severe myocardial dysfunction, except two who showed clinical and echocardiographic improvement (see relapsing type below).

## Group 2: non-compaction with congenital heart disease

**Fig. 3.**
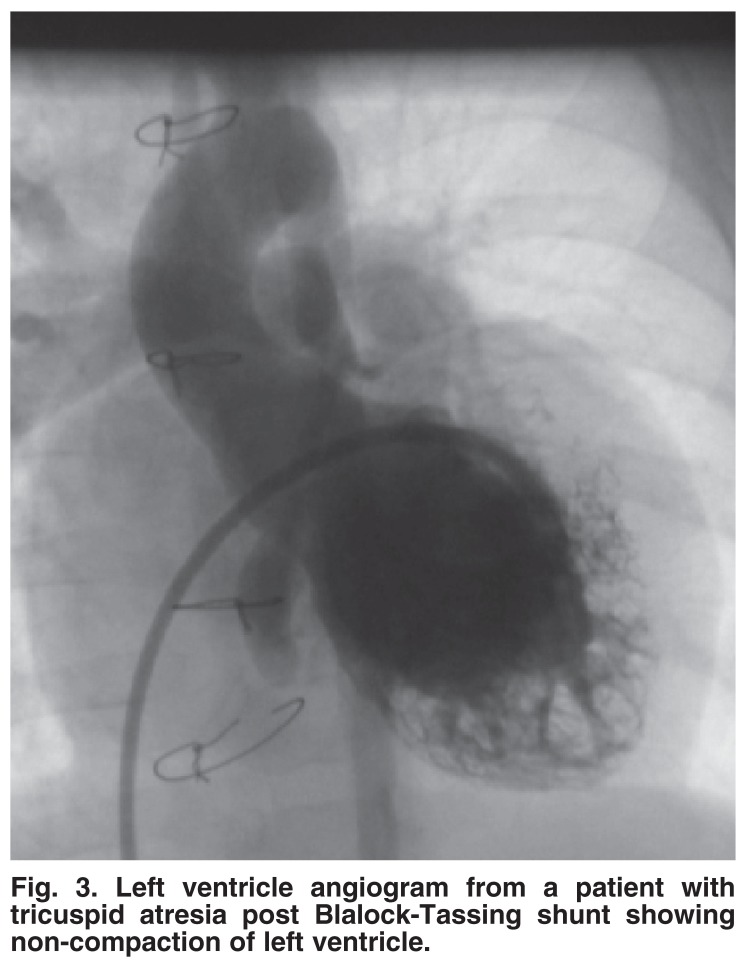
Left ventricle angiogram from a patient with tricuspid atresia post Blalock-Tassing shunt showing non-compaction of left ventricle.

A total of 26 patients had NCVM associated with CHD (Table 2). Complex heart defects were detected in 14 patients and non-complex defects in 12. Non-complex CHD included VSD in nine patients (two were associated with mitral valve cleft), patent ductus arteriosus (PDA), coarctation of the aorta and sub-aortic membrane, each in one patient. Complex CHD included pulmonary atresia (five patients), hypoplastic left heart with right ventricle NCVM (three patients), left atrial isomerism (three patients), tricuspid atresia (two patients) (Fig. 3), and double-outlet right ventricle (one patient). The clinical picture was dictated by the associated CHD. Four patients had Down syndrome. Those with simple CHD tended to have either normal or mildly impaired myocardial function while those with complex CHD had more extensive NCVM (which involved both ventricles in one patient) with severe myocardial dysfunction. Seven patients had surgery: VSD closure in four patients (two of whom had mitral cleft repair) and sub-aortic membrane resection in one patient.

**Table 2:** Clinical Features Of Patients With Non-Compaction With Congenital Heart Disease (N = 26)

Age in months range (mean)	0−240 (32)
Male/female ratio	1:1
Asymptomatic (%)	4 (15)
Down syndrome (%)	4 (15)
Simple CHD (%)	12 (46)
Complex CHD (%)	14 (54)
Myocardial dysfunction	16 (61)
Surgery (%)	7
Post-operative complications (%)	4 (80)
Death (%)	5 (19)
Improvement of myocardial dysfunction (%)	1 (3)

Post-operative complications included death due to ventricular tachycardia (one patient), large residual VSDs (two patients, one of whom had residual mitral cleft), ventricular fibrillation (one patient), and myocardial dysfunction (one patient). In this group, five patients died. All had complex CHD; one with pulmonary atresia and atrioventricular septal defect, who died with ventricular arrhythmia immediately post Blalock-Taussig shunt insertion, one patient with tricuspid atresia died suddenly one year after Blalock-Taussig shunt insertion and the others (hypoplastic left heart) died without surgery. The presence of NCVM affected surgical decisions in seven patients. One patient with coarctation of the aorta improved clinically and echocardiographically (see relapsing type below).

## Group 3: non-compaction with mitral regurgitation

**Fig. 4.**
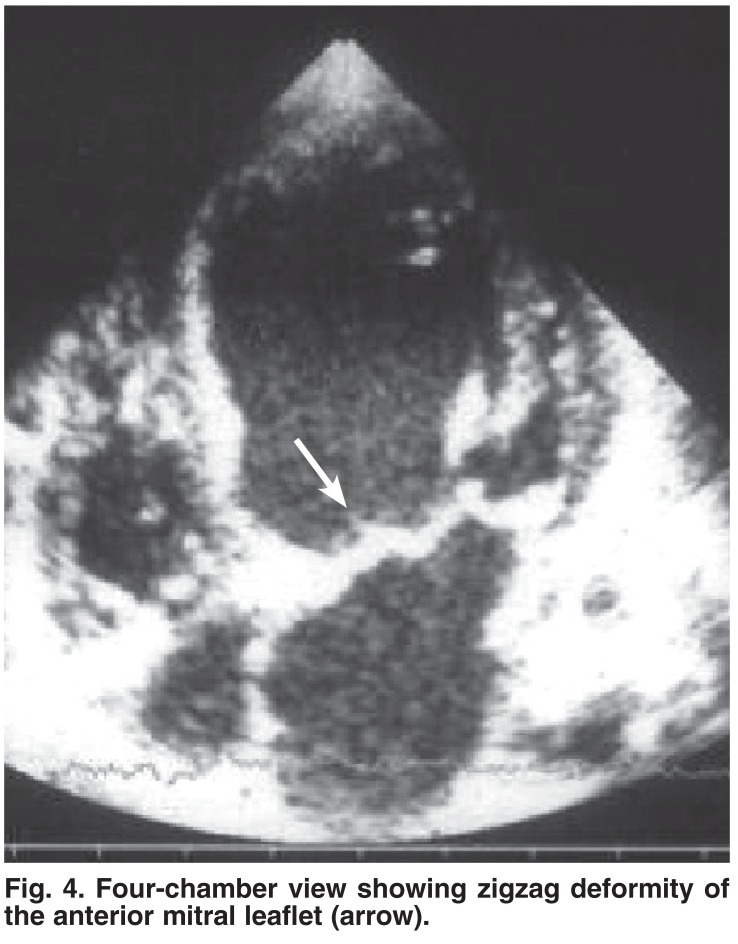
Four-chamber view showing zigzag deformity of the anterior mitral leaflet (arrow).

Seven patients had associated mitral valve pathology (Table 3). Six patients had isolated NCVM and one had associated VSD (included in group 2). Mitral regurgitation was associated with mitral valve deformity in the form of malcoaptation and a zigzag appearance of the anterior mitral valve leaflet (Fig. 4). The female-to-male ratio was 6:1. Mitral regurgitation was mild in one patient, moderate in three and severe in three. Associated abnormalities included relapsing type of myocardial dysfunction in one patient, left ventricular hypertrophy in two and infective endocarditis with mitral valve vegetation in one. One patient had uncomplicated mitral valve replacement at the age of one year.

**Table 3:** Clinical And Echocardiographic Feautres Of Patients With Non-Compaction With Mitral Regurgitation

*Patient*	*Age*	*Gender*	*Clinical and echo features*	*Outcome*
1	12 months	female	Heart failure. Echo showed non-compaction with mitral valve deformity and severe regurgitation, ejection fraction 62%	Follow up 6 months, no change
2	8 years	female	Mild heart failure, left ventricle hypertrophy, mitral valve deformity and moderate regurgitation, no systolic anterior motion of mitral valve. Infective endocarditis with a mitral valve vegetation, ejection fraction 80%	Continued to be asymptomatic with moderate MR
3	12 years	female	Asymptomatic. Non-compaction with mitral valve deformity and moderate regurgitation, ejection fraction 65%. Left ventricle dilated to 5 cm.	Continued to be asymptomatic with moderate MR
4	6 months	female	Twin 1. Severe heart failure due to MR, preserved function. Mitral valve replacement at one year of age	Heart failure improved after MV replacement
5	2 months	female	Twin 2. Severe heart failure due to depressed function, mild MR	Heart failure and ventricular function improved over 3 months. Left ventricular hypertrophy appeared after improvement of LV function. MR remained mild
6	12 months	male	Heart failure due to severe MR, ejection fraction 65%	Continued the same
7	1 year	female	Large VSD, moderate MR	Residual VSD patch leak and moderate MR

## Relapsing type

Four patients (all females) showed improvement of their myocardial dysfunction during the course of follow up (Table 4). Two patients had isolated NCVM, one had mitral valve regurgitation and one had associated coarctation of the aorta. In one patient there were two relapses and remissions. In this patient the first presentation was at the age of seven years, during which she developed heart failure needing hospitalisation for two weeks, then complete recovery ensued and continued for seven years. The second episode also took two weeks to resolve but she developed a cerebrovascular accident (see above). Other patients showed one remission: two with complete recovery of heart failure and ejection fraction and one with marked improvement but not complete resolution.

**Table 4:** Clinical And Echocardiographic Features Of Patients With Relapsing Type Of NCVM

*Patient*	*Age*	*Gender*	*Clinical and echo features*	*Outcome*
1 (included in Table 3 above)	2 months	female	Twin 2. Severe heart failure due to depressed function, mild MR	Heart failure and ventricular function improved over 3 months. Left ventricular hypertrophy appeared after improvement of LV function. MR remained mild
2	4 months	female	Heart failure. Dilated left ventricle with ejection fraction of 15%	Followed up for 18 months. Heart failure decreased and ventricular function improved to 40%
3	2 months	female	Heart failure needing hospital admission. Coarctation of the aorta with a gradient of 40 mmHg. Low ejection fraction of 40%	Heart failure improved over 2 months. Ejection fraction improved to 60%. Waiting for coarctation repair
4	15 years	female	Heart failure with history of similar condition 7 years before. Transient hemiparesis. Echo: ejection fraction 25%, LV thrombus that resolved with anticoagulation	Improved clinically over 6 weeks. Ejection fraction improved to 60%. Splenic haematoma secondary to warfarin

## Statistical analysis

Fischer’s exact test was used to compare the groups. Females in the group with isolated NCVM were significantly more than in the group with associated CHD (*p* = 0.03, odds ratio = 4.2, 95% CI = 0.529−16.1). Ejection fraction, improvement of function and mortality were not statistically different between the patients with isolated NCVM and those with associated CHD. For the ejection fraction: *p* = 0.226, odds ratio = 2, 95% CI = 0.557−7. For improvement of function: *p* = 0.419, odds ratio = 2.63, 95% CI = 0.222−31.2. For mortality: *p* = 0.307, odds ratio = 0.442, 95% CI = 0.077−2.55. Patients with associated MR were not compared with the others because of the small sample size.

## Discussion

Non-compaction of the ventricular myocardium is a disease with variable presentations and outcomes. It is still placed under ‘unclassified cardiomyopathies’ in the World Health Organisation classification, indicating the need for more investigations to clarify its anatomical−echocardiographic−clinical correlations. However, reliable diagnostic criteria are available and using these strict criteria we managed to identify a frequency of 22 in 10 000 echocardiograms in KSA as well as in Sudan. Lower but relatively high frequencies were also reported from the United States of America and from Australia, where the disease was thought to be relatively common.^6^,^7^

We have no doubt, therefore, that this disease is underdiagnosed and that the increased awareness of echocardiographers reveals more and more cases. Moreover, many cases are detected on routine screening in asymptomatic individuals, and parents and siblings of affected children, as in our patients, indicating that the true incidence is probably under-estimated. This is also evident from the high number of patients with Down syndrome in our series, as such patients are routinely screened by echocardiography.

Important clinical and echocardiographic features were noted in this cohort, which is the largest, to our knowledge, from the Arab/African population. Clear female predominance in isolated NCVM and that associated with mitral regurgitation is unique to this area of the world, in contrast to other reports of male predominance.^6^,^7^ We did not encounter any patients with Barth syndrome (cardiomyopathy, neutropenia and growth deficiency associated with deletion of Xq-28 in males) which was previously found to be prevalent in patients with NCVM.^8^ Familial NCVM was present in almost a third of our patients. In many families, more than two siblings were affected, with a lethal outcome in six children, who were not included in the study.

There is a need, therefore, for genetic studies, as NCVM in our area seems to be genetically distinct from that in other areas. We are currently planning to carry out such studies in collaboration with other centres. Many studies have recently shown that the genetics of NCVM is quite heterogeneous and diverse.^9^

Remission of myocardial dysfunction in patients with NCVM was first reported by us,^10^ and created some debate as to whether NCVM could be a transient response of the myocardium to stress. Subsequently, Pignatelli *et al.*^7^ reported other cases. In this series, four further cases are reported, which confirm that relapses and remissions are unique features of NCVM. One patient had two relapses and two remissions, with complete resolution of the myocardial dysfunction. This feature is one of the few clinical signs that can distinguish NCVM clinically from other types of cardiomyopathy. In all patients the myocardial trabeculations and recesses continued to be seen during the remission but, interestingly, the myocardium became hypertrophied in one patient during the remission. This observation was similar to that of Pignatelli *et al.*,^7^ which they referred to as ‘undulating phenotype’. This may correlate with another important feature of NCVM in one of our patients (mitral regurgitation group) who had left ventricular hypertrophy and normal/high ejection fraction, and raised the question of whether this patient was in remission.

The association of NCVM with mitral valve pathology is consolidated in this report by the addition of three more patients to our previously reported cases.^1^ All patients manifested characteristic MV changes and there was no other pathology to explain the presence of mitral regurgitation. We strongly feel that this association is unique and patients with mitral valve disease as well as those with NCVM should be carefully screened.

The association of NCVM with neuromuscular disorders is well documented.^11^ In one family in our series, all patients (total number five, all females) died with severe hypotonia and myocardial dysfunction in infancy. This family needs genetic work-up and further neurological investigations such as muscle biopsy, which we had planned to do before the death of the index case.

In our series, we were guarded about the short-term outcome of patients with NCVM, although many patients did not deteriorate, but a mortality rate of 9% in patients with isolated NCVM and up to 19% of those with CHD was detected. In asymptomatic patients and those with a normal ejection fraction, the short-term prognosis seemed to be more favourable. More important was the strikingly high frequency of post-operative complications in the form of ventricular arrhythmias and significant residual lesions.

NCVM can therefore have important implications on surgery and it should be taken into account when discussing these patients with the surgeon. In patients with complex lesions needing multiple-stage repair/palliation, the presence of NCVM can be a limiting factor for surgery and its presence has caused some patients to be dropped from surgical lists in our cohort, especially when the ventricular function was impaired.

## Conclusion

We have described the largest series of Arab and Arab/African patients with NCVM where the following unique clinical and ehocardiographic features were demonstrated: female predominance, relapsing course, left ventricular hypertrophy and mitral valve regurgitation, and a guarded short-term outcome. Genetic work-up is highly needed in this area.

## References

[1] Ali SKM, Omran AS, Najm H, Godman MJ (2004). Noncompaction of ventricular myocardium with mitral regurgitation and preserved ventricular function.. J Am Soc Echo.

[2] Ali SKM, Godman MJ (2004). The variable clinical presentation of, and outcome for, noncompaction of the ventricular myocardium in infants and children, an under-diagnosed cardiomyopathy.. Cardiol Young.

[3] Pignatelli RH, McMahon CJ, Dreyer WJ, Denfield SW, Price J, Belmont JW (2003). Clinical characterization of left ventricular noncompaction in children: a relatively common form of cardiomyopathy.. Circulation.

[4] Jenni R, Oechslin E, Schneider J, Jost CA, Kaufmann PA (2001). Echocardiographic and pathoanatomical characteristics of patients with isolated left ventricular non-compaction: a step towards classification as a distinct cardiomyopathy.. Heart.

[5] Oechslin EN, Attenhofer Jost CH, Rojas JR, Kaufmann PA, Jenni R (2000). Long-term follow up of 34 adults with isolated left ventricular noncompaction: a distinct cardiomyopathy with poor prognosis.. J Am Coll Cardiol.

[6] Pignatelli RH, McMahon CJ, Dreyer WJ, Denfield SW, Price J, Belmont JW (2003). Clinical characterization of left ventricular noncompaction in children: a relatively common form of cardiomyopathy.. Circulation.

[7] Nugent AW, Daubeney PE, Chondros P, Carlin JB, Cheung M, Wilkinson LC (2003). The epidemiology of childhood cardiomyopathy in Australia.. N Engl J Med.

[8] Bleyl SB, Mumford BR, Brown-Harrison MC, Pagotto LT, Carey JC (1997). Xq28 – linked noncompaction of the left ventricular myocardium: prenatal diagnosis and pathological analysis of affected individuals.. Am J Med Genet.

[9] Zaragoza MV, Arbustini E, Narula J (2007). Noncompaction of the left ventricle: primary cardiomyopathy with an elusive genetic aetiology.. Cur Opin Pediatr.

[10] Ali SKM, Du Plessis J, Godman MJ (2002). Noncompaction of the ventricular myocardium in infants, extended spectrum of a ‘specific’ cardiomyopathy.. Heart.

[11] Finsterer J, Stollberger C, Blazek G (2006). Neuromuscular implications in left ventricular hypertrabeculations/noncompaction.. Int J Cardiol.

